# CONCEPTUAL ADVANCEMENT FOR SOCIAL HEALTH IN DEMENTIA RESEARCH

**DOI:** 10.1093/geroni/igac059.1705

**Published:** 2022-12-20

**Authors:** Myrra Vernooij-Dassen, Eline Verspoor, Marieke Perry, Karin Wolf-Ostermann

**Affiliations:** Radboud University, Nijmegen, Gelderland, Netherlands; Radboud University, Nijmegen, Gelderland, Netherlands; Radboud University, Nijmegen, Gelderland, Netherlands; University of Bremen, Bremen, Bremen, Germany

## Abstract

The lack of conceptual clarity on social health in dementia research hinders its articulation. We aim to apply concept advancement for social health to provide conceptual clarity by building from a conceptual meaning to domains. The procedure is underpinned by theoretical models and epidemiological evidence on the relation between social heath and cognitive functioning.This led to considering social health as a reciprocal relational concept that refers to the influence that an individual has on others (social environment), and vice versa. We distinguished three domains defining the individual level, representing the social competences of the individual, and three domains defining the social environmental level (structure , function and appraisal of the relationship. We hypothesize that social health acts as a driver for stimulating the use of cognitive reserve. This conceptual advancement promotes developments that integrate neurobiological and social sciences and new interventions to support older people with and without dementia.

